# TRAQ in clinical practice: A narrative review for health care transition readiness

**DOI:** 10.1016/j.hctj.2026.100152

**Published:** 2026-09-13

**Authors:** Betsy Hopson, Kylee R. Phalen, David Wood

**Affiliations:** a Department of Urology, University of Alabama at Birmingham, Birmingham, AL, United States; b Department of Social Work, East Tennessee University, Quillen College of Medicine, Johnson City, TN, United States; c Department of Pediatrics, East Tennessee University, Quillen College of Medicine, Johnson City, TN, United States

**Keywords:** Health care transition, Transition readiness, Transition Readiness Assessment Questionnaire, Adolescents and young adults, Motivational Interviewing

## Abstract

**Background:**

Advances in medical care have dramatically improved survival rates for children and youth with special health care needs (SHCN), with more than 90% now reaching adulthood. As a result, pediatric-to-adult health care transition has become a critical component of lifespan care. Despite national consensus recommendations, fewer than one in four adolescents receive the transition services needed to prepare them for adult-oriented care. Standardized, validated instruments to assess and promote transition readiness are essential to close this gap.

**Objective:**

This review describes the practical application of the Transition Readiness Assessment Questionnaire (TRAQ) in ambulatory care settings, to guide clinicians in its administration, interpretation, and integration with evidence-based counseling strategies.

**Methods:**

This narrative review synthesizes published literature and the authors' collective clinical experience implementing the TRAQ across ambulatory transition programs. Sources include the three successive TRAQ validation studies, national consensus guidelines, and behavior change literature underpinning the instrument's theoretical framework. The Transtheoretical Model of Behavior Change and Motivational Interviewing are presented alongside a structured clinical framework for iterative assessment, goal-setting, and follow-up.

**Results:**

The TRAQ is a validated, theory-driven instrument spanning four behavioral domains of health and health care self-management. It supports providers in systematically evaluating readiness, identifying skill gaps, and facilitating self-management development across adolescence. Practical guidance is provided for score interpretation, stage-matched counseling approaches, and a TRAQ Companion Individualized Transition Plan (ITP) for structured goal documentation and follow-up.

**Conclusions:**

Systematic use of the TRAQ within a structured transition counseling framework enables clinicians to evaluate readiness and tailor interventions to each youth's stage of change. Integration of the TRAQ and its companion ITP into routine ambulatory practice represents an evidence-informed approach to improving health care transition outcomes for adolescents and young adults (AYAs) with SHCN.

## 1 Introduction

According to the 2022–2023 National Survey of Children's Health, more than 1 in 4 children (26.2%) in the United States have special health care needs (SHCN).^1^ Advances in health care have improved the survival of children with SHCN, with more than 90% now reaching adulthood.^2^^,^^3^ As a result, the transition from pediatric to adult care has become a critical component of lifespan health care.^4^ Health care transition (HCT) has been defined as “purposeful, planned movement of adolescents and young adults with chronic physical and medical conditions from child-centered care to an adult-oriented health care systems”.^5^ Despite this recognition, national surveys indicate that only 21.7% of all adolescents (including those with SHCN) ages 12–17 receive the services necessary to prepare for transitioning to adult health care.^6^ Those services include a provider discussing changing to an adult health care provider, encouraging youth self-management (e.g., taking medication, talking to doctors), and helping develop a transition plan.

Many adolescents and young adults (AYAs) struggle to adapt to the increased autonomy required for disease or health self-management and for interacting with providers independently as they leave pediatric care.^4^^,^^7^, ^8^, ^9^, ^10^ Studies have demonstrated that many AYAs with SHCN have not developed the general or disease-specific self-management skills necessary for successful transition, resulting in gaps in care and declines in health outcomes.^8^^,^^10^^,^^11^ Recent consensus clinical reports and longitudinal studies of HCT across multiple conditions have identified robust transition planning and the promotion of “transition readiness”, defined as the acquisition of self-management skills enabling youth to manage their own health and health care needs, as key indicators of successful HCT.^2^^,^^8^

As defined by national consensus guidelines, transition readiness assessment encompasses the ongoing evaluation and promotion of health and health care self-management skills across several key domains: understanding one's health needs (both disease-specific and preventive), mastering self-management activities, navigating the health care system (including scheduling appointments and managing medications or medical equipment), and understanding and maintaining health insurance.^2^ Integration of standardized readiness assessment instruments, such as the Transition Readiness Assessment Questionnaire (TRAQ), into routine clinical care beginning at age 12 is recommended,^2^ and higher transition readiness has been associated with improved clinical outcomes, including fewer disease relapses and hospitalizations, better treatment adherence, and successful transfer to adult care. 12, 13, 14

The TRAQ was developed by our team across three successive iterations, each building upon prior psychometric and clinical evidence.^15^, ^16^, ^17^ The instrument has since been adopted across diverse clinical settings and patient populations, adapted for diagnosis-specific use, and employed as both a process and outcome measure in numerous research projects examining HCT.^12^^,^^18^, ^19^, ^20^, ^21^, ^22^, ^23^, ^24^ Having received numerous requests from clinicians for practical guidance on operationalizing the TRAQ, the authors present this review to describe the theoretical underpinnings of the instrument, provide guidance on its administration and interpretation, and outline evidence-informed counseling strategies for promoting transition readiness in clinical settings.

## 2 Methods

As a narrative review, this article did not employ a systematic review protocol. Sources were purposively selected based on the authors' roles as developers of the TRAQ and their collective experience implementing it in clinical practice, and included the three successive TRAQ validation studies, national consensus guidelines on health care transition, and foundational behavior change literature. Purposive selection was supplemented by a structured PubMed search conducted August 25, 2026, using the following search sequence: ("Transition Readiness Assessment Questionnaire"[Title/Abstract] OR "TRAQ"[Title/Abstract]) AND ("transition"[Title/Abstract] OR "transition to adult care"[MeSH Terms]). The search covered database inception through the search date with no language restrictions and returned 149 records, which the authors screened for psychometric evaluations, diagnosis-specific and transcultural adaptations, and clinical applications of the instrument; relevant articles identified through this process were incorporated into the review.

## 3 Theoretical framework

### 3.1 The transtheoretical model of behavior change

The TRAQ was developed within the theoretical framework of the Transtheoretical Model (TTM) of Behavior Change.^25^ Each item employs a 5-point Likert response scale corresponding to the five stages of change: Precontemplation, Contemplation, Preparation, Action, and Maintenance. Understanding these stages is essential for interpreting TRAQ scores and tailoring clinical interventions accordingly.

In the Precontemplation stage (score = 1), individuals do not intend to act in the foreseeable future and may be unaware that a skill deficit exists. Youth in this stage respond: “No, I do not know how.” The Contemplation stage (score = 2) is characterized by awareness of a need and active consideration of change, though without a commitment to act; youth at this stage indicate: “No, but I want to learn.” At the Preparation stage (score = 3), individuals intend to act imminently and have begun making small behavioral steps toward change; at this stage youth respond: “No, but I am learning to do this.” The Action stage (score = 4) involves active modification of behavior, typically sustained for less than six months; youth at this stage respond: “Yes, I have started doing this.” Finally, the Maintenance stage (score = 5) reflects consistent, sustained behavior change maintained for more than six months; youth at this stage respond: “Yes, I always do this when I need to.”

When administering the TRAQ, providers are encouraged to review these response levels aloud with the youth, confirm comprehension, and clarify definitions as needed. This practice establishes a shared language for behavior change and lays the foundation for productive goal-setting conversations throughout subsequent visits.

### 3.2 Motivational interviewing

Motivational Interviewing (MI) provides the complementary communication framework for translating TRAQ scores into behavior change. MI engages youth by meeting them at their current stage of readiness, affirming their autonomy, and evoking and supporting autonomous motivation. Youth at different stages of the TTM require tailored counseling strategies informed by MI principles. Clinicians are encouraged to consult established MI training resources and to apply MI techniques consistently across transition encounters.^26^, ^27^, ^28^

## 4 The TRAQ: instrument overview and psychometric properties

### 4.1 Domains and item content

The current version of the TRAQ (version 6.0; see Appendix 1) comprises items across four empirically derived domains, each reflecting behaviors, rather than knowledge, central to independent health and health care self-management. The focus on observable behaviors reflects a deliberate theoretical choice to operationalize the TTM within the instrument and does not preclude the parallel provision of disease-specific health education. The four domains are:

Managing Medications: Items addressing the ability to obtain or reorder prescriptions, communicate with pharmacists regarding medication use, drug interactions, and side effects.
Appointment Keeping: Items covering the ability to schedule appointments, follow up on referrals or test results, arrange transportation, and contact the provider regarding changes in health status.
Tracking Health Issues: Items related to completing medical history forms, maintaining a calendar or medication list, communicating health concerns to the provider, participating in medical decision-making, and attending portions of appointments independently.
Talking with Providers: Items assessing the ability to ask and answer clinical questions, request clarification, describe one’s health history, and communicate adherence to the care plan.

Domain content and item phrasing were developed through iterative input from HCT providers and youth. Across its three iterations, the TRAQ has demonstrated strong internal consistency at the total and domain level, with its factor structure confirmed through exploratory and confirmatory factor analysis in large samples of adolescents and young adults with diverse special health care needs.^15^, ^16^, ^17^ A systematic review of transition readiness instruments identified the TRAQ as among the most extensively validated measures available.^29^ Independent validations have extended this evidence to additional populations, including adolescents and young adults with inflammatory bowel disease^30^ and youth receiving mental health services.^31^ Evidence for predictive validity is emerging: higher TRAQ scores have been associated with successful transfer to adult care in sickle cell disease,^13^ with improved clinical outcomes including fewer relapses and hospitalizations in inflammatory bowel disease,^12^ and with clinical outcomes among youth with spina bifida using the TRAQ-SB.^14^ The TRAQ has also been adopted internationally, with validated translations and transcultural adaptations including Japanese,^32^ Brazilian Portuguese,^33^ Argentine Spanish,^34^ and Mexican Spanish,^22^ among others, supporting its use across health systems and cultural contexts. Written at a 6th-grade reading level, the TRAQ is designed to be accessible across a broad range of literacy levels. The instrument also includes two supplemental questions assessing the youth’s perceived importance of developing self-management skills and their confidence in doing so, constructs aligned with the MI framework of importance and self-efficacy.

### 4.2 Administration and frequency

The TRAQ is designed for repeated administration over time, ideally every six months and at minimum annually, beginning at age 12–14. Administration within a transition counseling visit, which, with sufficient practice, can be accomplished in 10–15 min, is readily integrable into busy ambulatory practices when responsibilities are distributed across clerical and clinical staff.^35^ Ideally, youth should complete the instrument independently and without parental assistance to obtain the most accurate self-report; however, parental input may be used to verify or revise reported scores during the review phase.

When a youth’s developmental or literacy level precludes independent completion, the instrument may be administered verbally by a staff member, or a caregiver may complete it on the youth’s behalf, based on the youth’s demonstrated abilities. For youth with complex physical or cognitive limitations who will require ongoing assistance with self-management tasks, the caregiver and youth may be considered a functional “team,” and the assessment completed jointly to evaluate the team’s collective competence in transition-relevant activities. Prior TRAQ scores should be reviewed at each subsequent visit to confirm progress or identify persistent areas where improvement can be encouraged.

## 5 Clinical implementation framework

### 5.1 Introducing health care transition in early adolescence

**Fig. 1:**
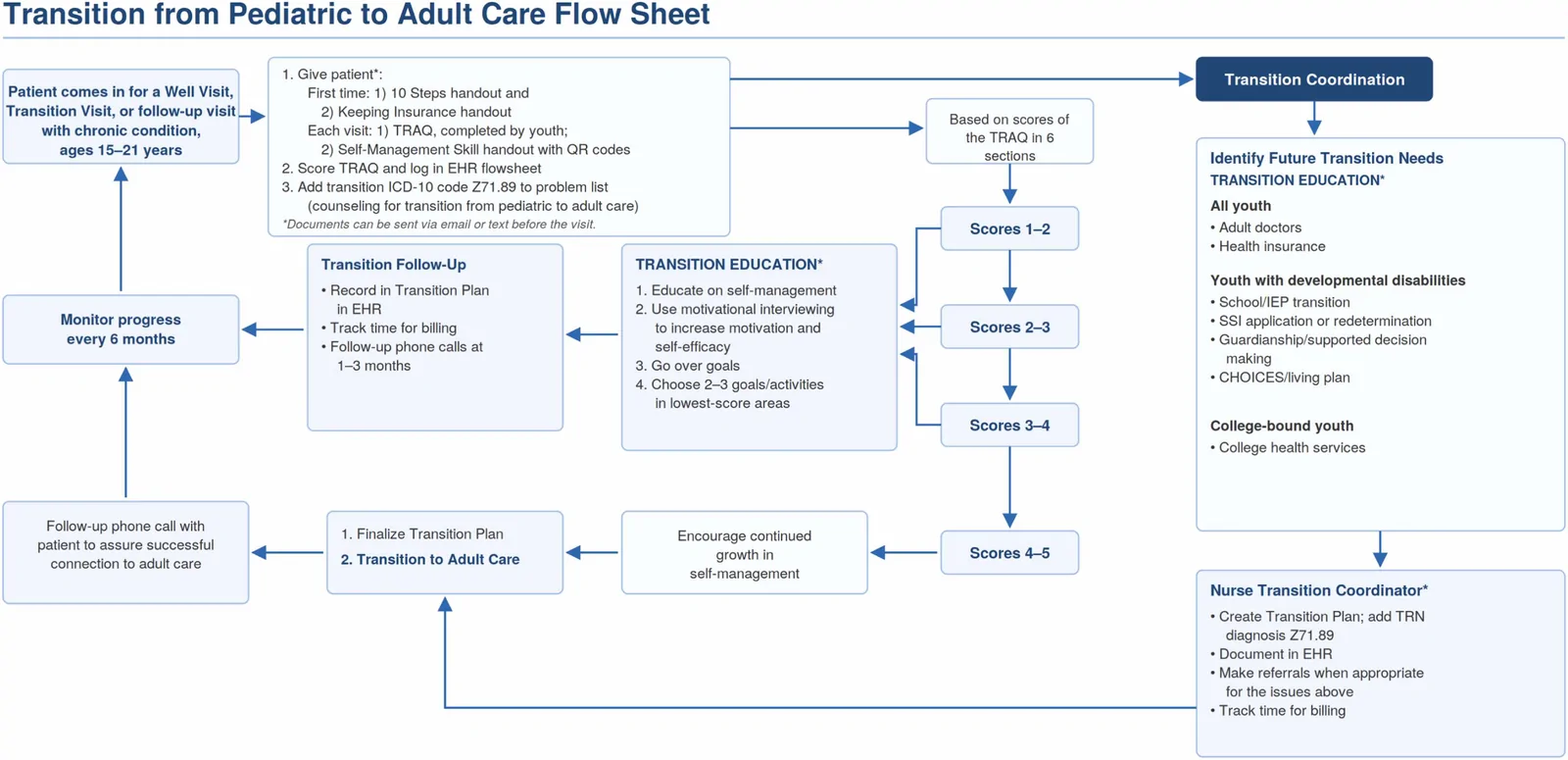
Iterative TRAQ Clinical Workflow. Stepwise workflow guiding clinicians through transition readiness assessment, stage-matched counseling, and individualized goal-setting across ambulatory visits, repeating iteratively as the youth progresses through adolescence. TRAQ = Transition Readiness Assessment Questionnaire; EHR = electronic health record.

Between ages 12 and 14, providers should begin introducing HCT as a routine topic at each encounter. Initial transition education includes orienting youth and their families to the concept of health care transition, providing foundational resources (e.g., informational materials from Got Transition), and introducing the TRAQ. Providers should explain the instrument’s four domains, review the five response categories and their behavioral meanings, and invite the youth to complete the instrument independently. In the authors' practice, responsibilities are distributed to minimize provider burden: clerical or clinical support staff distribute the TRAQ at check-in, and youth complete it in approximately 5 min; a nurse, medical assistant, or transition coordinator scores the instrument in 1–2 min; and the provider or transition coordinator reviews results, facilitates goal-setting, and documents the plan within the 10–15 min counseling encounter described above. Post-visit telephone or portal follow-up, typically conducted by the transition coordinator or clinical staff, requires approximately 5–10 min per patient. Specific role assignments will vary by setting; in practices without a dedicated transition coordinator, these functions may be shared across existing nursing and clinical support staff. Fig. 1 presents the iterative clinical workflow used by the authors to guide the ongoing assessment, education, and goal-setting process. The center-top box starts the process with giving out two handouts: an overview of the transition process (10 steps) and information about maintaining insurance during transition. These handouts can be found at www.floridahats.org but should be adapted for local use. The TRAQ is given at this step in the process.

### 5.2 Interpreting TRAQ scores and guiding goal-setting

TRAQ scores are most meaningfully interpreted in longitudinal context, analogous to monitoring growth on a developmental growth chart.^36^ The strongest predictor of TRAQ scores is age, reflecting the cognitive developmental transition from concrete to formal operations that enables forward planning and new skill acquisition. Targeted interventions to promote self-management skills, in addition to developmental maturation, are associated with improvements in TRAQ scores over time.^12^^,^^17^^,^^37^, ^38^, ^39^, ^40^, ^41^

When youth consistently achieve average domain scores of 4.0 or higher, this indicates readiness to assume full responsibility for health and health care self-management and supports a transition to adult-oriented care. The following guidance is offered for each scoring range:

• For youth with average domain scores of 1–2 (Precontemplation to early Contemplation), the provider should explore the youth’s broader life goals and identify how self-management skill development supports those aspirations (e.g., driving, attending college, employment). Goals should be modest, achievable, and broken into small steps, for example, watching a brief instructional video about a target skill.
• For youth scoring in the 2–3 range (Contemplation to early Preparation), providers should begin by affirming what the youth is already doing, then collaboratively identify a single additional step toward a new goal. As the youth demonstrates readiness for further development, the provider may shift toward a coaching stance (e.g., “That’s a great idea, let’s talk about how to make it happen.”).
• For youth scoring 3–4 (Preparation to Action), the provider assumes a full coaching role. The focus at this stage is on reinforcing prior accomplishments and encouraging the youth to pursue new goals that extend independence across all TRAQ domains.

### 5.3 Using the importance and confidence scales in building self-efficacy

Before moving to skill-building, clinicians should assess the two supplemental TRAQ questions on importance and confidence. These items are critical to interpreting the clinical utility of the domain scores and should inform the counseling approach before any goals are established. Importance reflects how much the youth values developing self-management skills; confidence reflects their perceived self-efficacy in doing so. A youth who rates importance low may require values-based exploration, consistent with MI’s emphasis on eliciting autonomous motivation, before skill-building can meaningfully begin. If the youth is cognitively immature or resistant to taking responsibility, a structured incentive system developed collaboratively with the caregiver may be appropriate. A youth who rates confidence low, by contrast, is already motivated but needs encouragement, stepwise goal progression, and opportunities to rehearse skills in low-pressure contexts before attempting them in clinical settings.

This distinction matters because self-efficacy, defined as the belief in one's own capacity to execute a specific behavior, is the proximate driver of behavior change.^42^ Bandura's Social Cognitive Theory identifies mastery experiences as the most powerful source of self-efficacy: success at a task builds confidence that transfers to related challenges, a dynamic in which success breeds success^.^^43^ Clinicians can channel this developmental momentum by framing TRAQ-guided goal-setting not as a checklist of health tasks to complete, but as a graduated progression of independence-building experiences.

Importantly, entry-level goals do not always need to be health-related. For example, a youth who scores low on "Talking with Providers" might begin by entering a restaurant alone and placing an order while the parent waits in the car. This allows the youth to practice self-initiated conversation with an unfamiliar adult without the emotional stakes of a clinical encounter. A youth working toward medication independence might first accompany a caregiver to the pharmacy as an observer before attempting the interaction independently. These non-clinical tasks build the same underlying capacities for communication, self-advocacy, and autonomous action that health care self-management requires.

Role-play can be a particularly effective complement for the communication domains of the TRAQ. Having a youth practice asking a provider a question, first with a parent in the provider role, then reversing, builds behavioral fluency before the skill is required in a real clinical setting. Role-play is valued specifically for the rehearsal and feedback it provides in realistic scenarios, allowing practice without real-world consequence.^44^

In practice, building self-efficacy within the TRAQ framework involves: first assessing importance and confidence to determine the appropriate counseling entry point; identifying one small, achievable goal tied to the lowest-scoring domain (while reinforcing areas of strength); using non-health care independence tasks as accessible entry points when clinical tasks feel out of reach; employing graduated steps before independent action; practicing clinical communication through role-play with parents or peers; and explicitly celebrating incremental success at follow-up visits, connecting each accomplishment to growing readiness for adult care. This scaffolded approach is well supported by the literature. As adolescents gain competence in progressively more complex self-management tasks, they assume greater responsibility for their health and health care.^45^

### 5.4 Applying motivational interviewing across TRAQ score levels

A particularly effective MI technique when reviewing TRAQ scores with a youth is to ask not, "why did you score yourself a 2?" but rather "why did you score yourself a 2 instead of a 1?" This reframing is deliberate: it invites the youth to articulate their own strengths and existing motivation rather than focusing on the gap between where they are and where they could be. The clinician is not pushing the youth toward a 3; they are celebrating that the youth sees themselves as a 2, meaning a desire to learn a new skill. This distinction is central to MI's spirit of affirmation and autonomy support. The same principle applies at any score level: asking why a youth rated themselves a 3 instead of a 2 surfaces the competencies they have already developed and builds the foundation for the next step.

Items scored at level 2 ("No, but I want to learn") represent particularly productive entry points for MI-based skill development, as they signal nascent motivation that is ready to be channeled. When a youth scores a 2 on appointment keeping, for example, the clinician might say: "I notice you aren't currently making your own appointments, but that you want to learn to do that, that’s exciting that you want to learn." From there, a concrete, achievable goal can be collaboratively constructed: the youth's next appointment becomes their first independent scheduling task. The youth might enter the date of their next appointment in their phone's calendar, set a reminder alert two weeks prior, and take on the goal of reminding their parent of the appointment. Each of these sub-steps is itself a small mastery experience that builds toward a score of 3.

This approach also connects the goal back to the youth's broader life aspirations, which MI identifies as powerful motivational anchors. A youth aspiring to attend college or live independently can be shown how pharmacy access, provider communication, and health history recall directly support those goals. A youth who wants to attend social events (e.g., a football game or dance) independently despite a chronic condition can use that aspiration as the motivational context for specific self-management targets. When the youth can see a direct line between a TRAQ skill and something they already care about, the goal shifts from a clinician-assigned task to a self-chosen step toward their own future, which is precisely where durable behavior change begins.

### 5.5 Skill-building activities by TRAQ item

Table 1 provides structured coaching activities and associated behavior change goals for each TRAQ item, intended to guide providers in translating scores into actionable clinical goals. Goal selection should prioritize activities that are personally meaningful to the youth; however, when a high-priority clinical need exists (e.g., medication adherence for a time-sensitive regimen), the provider should collaboratively direct goal selection toward that area. Parents and caregivers may be productively engaged in supporting the youth’s goal achievement while avoiding patterns of over-control or social loafing. More broadly, they play an active and evolving role throughout transition preparation. In early adolescence, caregivers often serve as primary partners in skill development: modeling behaviors, participating in role-play, structuring opportunities for practice, and providing encouragement and accountability between visits. As the youth demonstrates growing proficiency, the caregiver's role intentionally shifts from direct management toward supervised practice, and ultimately toward consultation, with the youth assuming primary responsibility and the caregiver remaining available as a safety net. Providers can support this progression by naming it explicitly with families, eliciting caregiver goals and concerns at each visit as incorporated in the ITP framework, and helping caregivers calibrate their involvement to the youth's demonstrated abilities rather than to habit or worry. For youth who will require ongoing support due to cognitive or physical limitations, the same progression applies within the caregiver-youth team model described above, with the goal of maximizing the youth's participation within the dyad rather than full independence.

**Table 1:** TRAQ skill-building activities and behavior change goals by item. For each of the 20 TRAQ items across four domains, example coaching activities and corresponding behavior change goals to guide providers in translating TRAQ scores into actionable clinical objectives.

**TRAQ Item**	**Example Task / Coaching Activity**	**Goal or Behavior Change**
1. Fill a prescription	Caregiver and patient call the pharmacy together or use app to request refill; add refill reminder to phone calendar one week before refill due	Learns how to independently refill medications
2. Handle a bad reaction	Role-play describing symptoms and identifying who to contact; create a 'What to do if…' reference card	Knows who to call and what to say if an adverse reaction occurs
3. Reorder medications	Review label for refill number and date; set reminder to text/call parent when supply is low	Develops proactive medication management habits
4. Explain medications to providers	Practice naming each medication, dose, and reason	Communicates medication details accurately
5. Speak with pharmacist	Practice asking one medication question at pharmacy; write question in phone notes beforehand	Builds confidence initiating communication with pharmacist
6. Call doctor to make appointment	Practice scheduling next visit by phone; enter date into calendar with 2-week reminder	Learns to make and track appointments independently
7. Follow up on referrals	Review After Visit Summary for referral contacts; role-play calling to schedule	Completes referrals and follows through on next steps
8. Arrange transportation	Discuss transportation options; add route or contact to phone navigation	Plans and arranges rides to appointments
9. Call about unusual changes	Save nurse line and after-hours number in phone; role-play explaining symptoms	Initiates appropriate contact when new health issues arise
10. Fill out medical history form	Practice completing sample form; store digital copy in phone	Knows personal health history and can provide accurate information
11. Keep list/calendar of appointments	Set up recurring events and color-code health vs. personal appointments	Tracks appointments and responsibilities visually
12. Tell doctor what you feel	Practice describing symptoms (what, where, when, how bad); keep symptom diary in phone	Communicates symptoms clearly to providers
13. Contact doctor for concerns	Practice using patient portal or nurse line for questions; send first message together with provider	Uses health system communication tools appropriately
14. Help make medical decisions	Discuss pros/cons of two treatment options; reinforce that patient voice matters	Participates in shared decision-making
15. Attend part of visit alone	Set goal for 5 min of independent time in appointment	Builds independence and confidence with providers
16. Ask questions	Write one question before each visit; practice asking first	Develops self-advocacy and curiosity in health care
17. Answer provider questions	Role-play common questions; patient answers before parent	Responds independently and confidently
18. Ask for clarification	Practice saying 'Can you explain that another way?'	Learns to seek understanding when unclear
19. Follow up on advice	Summarize doctor's instructions aloud and message provider after completing step	Strengthens follow-through and accountability
20. Explain health history	Practice a 3-sentence health summary (diagnosis, surgeries, current care)	Gains confidence describing health background

## 6 Goal documentation using the individualized transition plan (ITP)

**Fig. 2:**
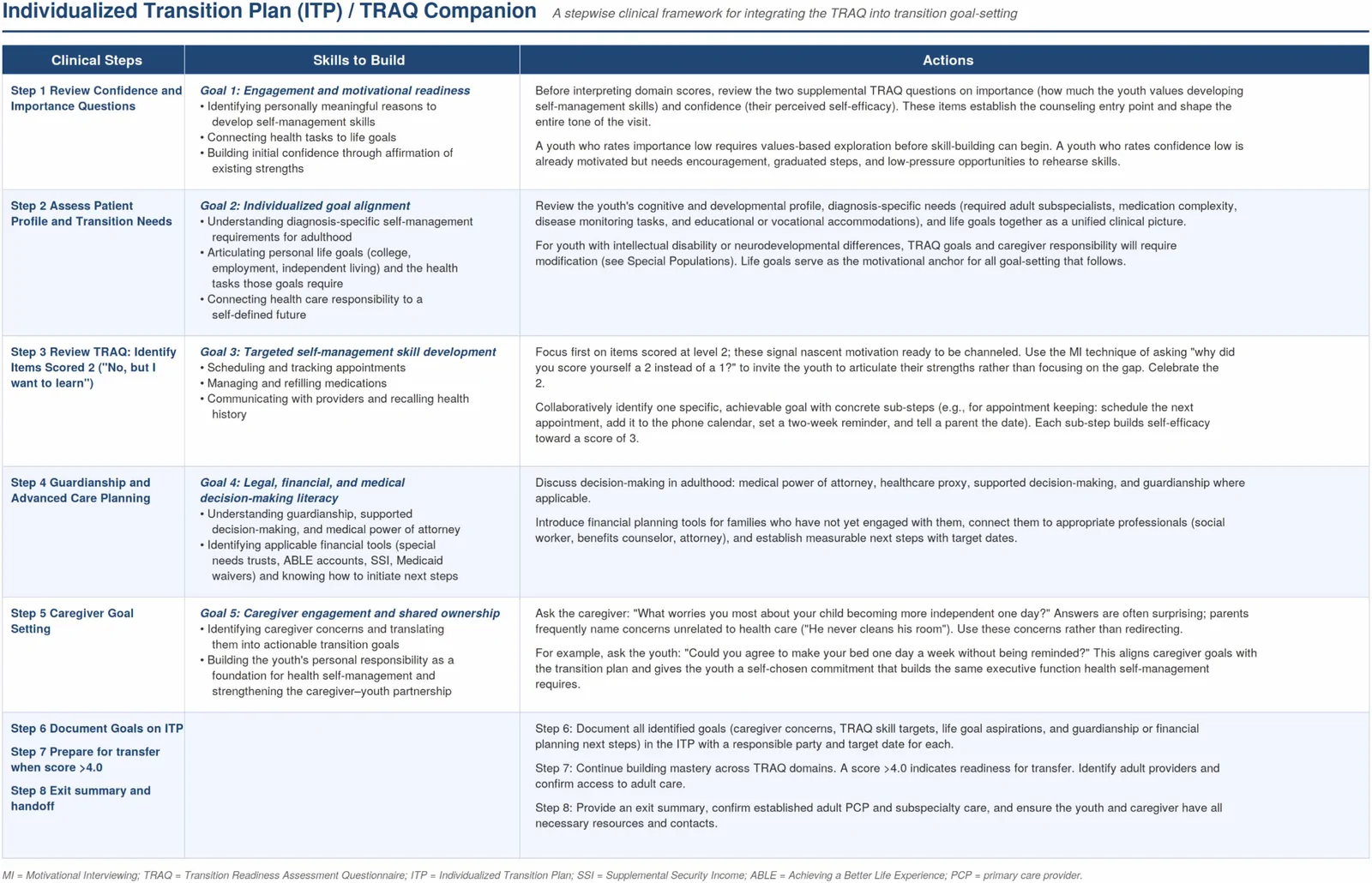
TRAQ Companion Individualized Transition Plan (ITP). Eight-step clinical framework guiding providers from initial TRAQ score review through goal documentation, progress monitoring, and confirmed handoff to adult care.

All transition goals agreed upon during the visit should be documented in the encounter note and carried forward into a structured Individualized Transition Plan (ITP).^36^^,^^38^ The TRAQ is most clinically useful when its results are translated directly into this kind of systematic, revisable document. A TRAQ Companion ITP, shown in Fig. 2, provides a stepwise clinical framework that guides the provider through eight sequential steps: reviewing the importance and confidence questions, assessing the youth's cognitive profile and diagnosis-specific transition needs, identifying TRAQ items scored at level 2, addressing guardianship and advanced care planning, eliciting caregiver goals, documenting all agreed-upon goals, monitoring progress toward a score of > 4.0, and completing an exit summary with confirmed handoff to adult providers.

The authors recommend presenting the documented goals as a collaborative agreement using accessible, affirming language: "I am going to document these goals, and I'd like you to review them with me. Think of this as a mutual agreement between us. Let's only include goals you actually intend to pursue." This framing reinforces the youth's autonomy and investment in the plan. Following the visit, the transition coordinator or clinical staff administering the TRAQ should contact the youth and, when appropriate, the caregiver by telephone or patient portal to review goal progress, address challenges, and facilitate next steps.

The frequency of follow-up should be calibrated to the youth's current TRAQ score range. Youth scoring in the 1–2 range benefit from early and more frequent outreach; those in the 2–3 or 3–4 range typically require less intensive contact as motivation and skill are already developing. Once a youth achieves scores of 4–5 across all domains, formal transition education may be de-escalated, though encouragement and monitoring should continue. At this stage, the provider and transition coordinator may initiate formal transfer to an adult care provider, including coordination with the receiving physician and submission of a referral.

The ITP provides a structured mechanism for documenting and tracking goals across all relevant transition domains, including health self-management, school and employment, insurance, family planning, safety, and other life areas. A blank fillable ITP template for use in clinical practice is included in Appendix 2.

### 6.1 Case illustration

The following case illustrates the application of TRAQ-guided transition counseling for a youth in early stages of self-management development.

John is a 15-year-old male with a chronic health condition presenting for a transition counseling visit. His TRAQ scores are as follows: total average 2.4; Managing Medications 1.8; Appointment Keeping 1.25; Tracking Health Issues 1.5; Talking with Providers 3.8; Importance 4.0, Confidence 2. When asked which domain he would like to prioritize, John initially selects Appointment Keeping (his lowest-scoring domain), though the transition coordinator suspects this reflects a desire to please rather than genuine interest. She redirects: “Let’s look at the Managing Medications section. It looks like you’re interested in learning those skills. Which one would you like to start with?” John selects filling a prescription and commits to watching an instructional video and then accompanying his mother to the pharmacy to speak with the pharmacist independently.

A telephone follow-up two months later reveals that John has viewed the video but has not yet spoken with the pharmacist. By his next visit six months later, John has completed the pharmacy interaction goal. His TRAQ is readministered, a new score is obtained, and the transition education cycle continues with updated goals aligned to his current developmental stage.

This case underscores several key principles of TRAQ-based counseling: the importance of attending to authentic rather than performed motivation; the value of small, achievable initial goals; the role of structured follow-up; and the iterative nature of the process as youth develop across adolescence.

## 7 Special populations

### 7.1 Youth entering college

For youth preparing to enter college, transition counseling should address several specific domains in addition to standard TRAQ-based skill development. These include anticipating changes in insurance coverage (particularly for those currently covered under a parent’s plan or Medicaid), discussing the transition to a new primary care provider, and planning for health care access in the college setting, whether through campus health services or community providers. Youth with disabilities should be encouraged to contact the campus disability services office prior to orientation to arrange appropriate accommodations. A comprehensive pre-college health care preparation checklist is provided in Appendix 3.

### 7.2 Youth with intellectual disabilities

Youth with intellectual disabilities require additional consideration of several transition-specific issues that extend beyond health care self-management. These include:

Reviewing options for and obtaining legal measures related to guardianship or health care surrogacy arrangements.
Navigating SSI Redetermination, which occurs at age 18 for all youth with disabilities and is a critical process for maintaining Supplemental Security Income and Medicaid health insurance coverage.
Supporting the Transition section of the Individualized Education Program (IEP), including planning for continued school enrollment through age 21 and transition to Vocational Rehabilitation programs.
Addressing self-determination issues related to living arrangements, including applications for Home and Community-Based Waivers that provide financial support for work and independent living.

Additional resources specific to this population, including state-level transition support services, are provided in Appendix 3.

## 8 Documentation and billing for transition counseling

Providers should document transition counseling encounters using the transition diagnostic code Z71.89 in the electronic health record following the initial transition counseling visit. This diagnosis code enables billing for time-based counseling related to the transition from pediatric to adult care. It may also be used as a registry identifier to track which patients have initiated the HCT process and to identify those due for follow-up. For youth requiring more frequent clinical monitoring, the TRAQ should be administered every six months. A detailed coding and reimbursement reference is available from Got Transition.^46^

## 9 Conclusion

The TRAQ is a validated, theory-driven instrument that enables clinicians to systematically assess and promote health care transition readiness in AYAs with special health care needs. Grounded in the Transtheoretical Model and Motivational Interviewing, it supports individualized, developmentally appropriate interventions that promote durable self-management skills. Providers caring for youth with complex or condition-specific needs should supplement TRAQ-based counseling with targeted disease-specific education.

The authors' clinical experience suggests that once youth engage with and commit to developing even one or two self-management skills, these behaviors tend to generalize across domains of health and health care management. The central elements of effective TRAQ-based practice (respectful engagement, genuine attunement to the youth's stage of readiness, and consistent use of motivational interviewing) are not merely clinical techniques but represent a relational approach to empowering adolescents to develop the skills they will carry through adulthood.

## Funding/Support

No funding was secured for this study.

## Role of Funder/Sponsor (if any)

N/A.

## Clinical Trial Registration (if any)

N/A

## CRediT authorship contribution statement

**Betsy Hopson:** Writing – review & editing, Writing – original draft, Supervision, Methodology, Formal analysis, Conceptualization. **Kylee R. Phalen:** Writing – original draft, Conceptualization. **David Wood:** Writing – review & editing, Writing – original draft, Project administration, Methodology, Conceptualization.

## Declaration of Competing Interest

The authors declare that they have no competing interests to disclose.

## Data Availability

No data was used for the research described in the article.
