# Supplementary material for: TRAQ in clinical practice: A narrative review for health care transition readiness
Source: Health Care Transit. 2026 Sep 13;4:100152. doi: 10.1016/j.hctj.2026.100152 (PMC13590068; doi:10.1016/j.hctj.2026.100152)
Supplement: Supplementary file 1 [file mmc1.docx]

Supplemental Files

Appendix 1: Transition Readiness Assessment Questionnaire- TRAQ

Appendix 2: ITP- TRAQ Guided Goal Setting

Appendix 3. Going to College Checklist

**Going to College Checklist**

*A guide for students with chronic health conditions preparing to leave for college*

Congratulations on going to college! Along with this exciting step comes new responsibility for managing your own health care. Use this checklist — with the help of your doctor or transition coordinator — to make sure you are ready before you leave.

*If you have concerns about any item on this list, please schedule a Transition Appointment before you leave for school.*

**SECTION 1: Managing Medications**

☐ Fill my own prescription at the pharmacy

☐ Reorder my prescription before it runs out

☐ Take my medications independently each day

*Use a pill organizer or phone alarm if needed*

☐ Know the name, dose, and purpose of each medication I take

☐ Know what side effects my medications can cause and who to call if I have one

☐ Speak with a pharmacist independently if I have a question

☐ Know what to do if I miss a dose

**SECTION 2: Keeping Appointments**

☐ Call and schedule a doctor's appointment on my own

☐ Arrange transportation to get to an appointment

*Bus route, rideshare, campus shuttle, or car*

☐ Attend a doctor's appointment independently (without a parent present)

☐ Keep a calendar of appointment dates and times

*Phone calendar, notes app, or planner*

☐ Know how to follow up on referrals or test results

☐ Know my insurance information and carry my insurance card

**SECTION 3: Tracking My Health**

☐ Explain my health history (diagnosis, surgeries, current medications, allergies)

☐ Complete a medical history form independently

☐ Contact my doctor or nurse for health concerns (phone, portal, or nurse line)

☐ Participate in medical decision-making during appointments

☐ Keep a list of my health care providers and their contact information

**SECTION 4: Talking with Providers**

☐ Ask questions at medical appointments without a parent present

☐ Answer provider questions independently and accurately

☐ Ask for clarification when I don't understand something

*"Can you explain that another way?"*

☐ Follow up on advice or instructions my provider gives me

**SECTION 5: Insurance & Local Health Care Access**

☐ Confirm whether my current insurance covers care in my college city

☐ Know how to maintain or obtain insurance if I am turning 26 or aging off a plan

*Visit healthcare.gov for marketplace options*

☐ Identified a primary care provider near my college or at the campus health center

☐ Know whether my college has a campus health clinic and what it offers

☐ If I have a disability, contacted the campus disability services office before orientation

*Request academic and housing accommodations early*

☐ If I take a controlled substance, know the regulations for having it on campus

**SECTION 6: If You Are Going Away to College**

☐ Found a specialist near my college if I need one (ask your transition team for a referral)

☐ Confirmed whether the university health center accepts my insurance or offers its own plan

☐ Arranged reliable transportation to off-campus health appointments

☐ Have a plan for obtaining my medications if I run out while away from home

☐ Know my after-hours or emergency contact number for my health condition

**Notes / Questions for my transition coordinator or provider:**

Appendix 4. Financial Planning for Young Adults with Disabilities

**Transition Planning for Young Adults with Disabilities**

*A guide to benefits, savings tools, and legal protections available to adults with childhood-onset conditions across the United States*

This guide covers federal programs and tools available in every state. Some sections note where state-level programs vary, your care team or social worker can help you find the specific programs in your state.

Key principle: Most benefits are based only on your own income and resources, not your parents' finances. You may qualify even if your family earns a good income.

**Step 1: Apply for SSI (Supplemental Security Income)**

When you turn 18, you can apply for SSI regardless of your parents' income. At 18, SSI eligibility is based only on your own income and resources - not your family's.

**What is SSI?**

- Monthly income provided by the federal government to help cover basic needs like food and housing.
- Available to adults 18+ with a disability who have limited income or savings.
- The income limit and asset limits are set federally but check with SSA for current figures.

**How to apply (same in every state):**

- Online: ssa.gov/ssi
- By phone: 1-800-772-1213 (Social Security Administration)
- In person: At your local Social Security office

Timing tip: Apply as soon as possible after your 18th birthday. SSI is not retroactive you only receive benefits from the date of your approved application, not from when you became eligible.

**Step 2: Understand Medicaid and Home & Community-Based Waivers**

Medicaid is a federal-state health insurance program for people with low income or disabilities. Every state runs its own Medicaid program, but all states offer Medicaid to adults with disabilities who meet the financial criteria.

**Home & Community-Based Services (HCBS) Waivers**

Medicaid waivers allow states to provide home- and community-based supports and services to people who might otherwise need to live in a facility. These can include personal care, nursing support, assistive technology, supported employment, and more.

- Waiver programs vary by state, each state has its own waiver types, eligibility criteria, and available services.
- Many waivers have waiting lists. Apply early, even if you don't think you need services immediately.
- You can be on the waiting list for one waiver while receiving services through another.
- Eligibility is based on the applicant's finances only, not the family's income.

**How to find waivers in your state:**

- Ask your care team or social worker if they can identify the right programs for your state.
- Visit medicaid.gov/medicaid/hcbs to find your state's waiver programs.
- Contact your state's Medicaid office directly - search "[your state] Medicaid waiver" or call 1-800-MEDICARE (1-800-633-4227) for referrals.
- Disability Rights organizations in your state can also help navigate the application process.

Common waiver types across many states include programs for: Intellectual/Developmental Disabilities (I/DD), Physical Disabilities, Technology-Assisted Care (e.g. ventilator-dependent), Elderly & Disabled Adults, and Community Transition.

Not every state has all of these, and names vary. Your social worker can identify which apply to you.

**❌ Common Myths — These statements are NOT true**

**✗** My parents earn too much -I won't qualify. (Eligibility at 18+ is based only on YOUR finances.)

**✗** There's an IQ requirement for all disability waivers. (Many waivers have no IQ requirement.)

**✗** You have to be on Social Security to get a Medicaid waiver.

**✗** Waivers are only for people who don't have private insurance.

**✗** If I'm already on Medicaid, I can't apply for a waiver.

**✗** Qualifying for a nursing-facility level of care means I have to live in a nursing home. (It does not — it is a documentation standard, not a placement decision.)

**Step 3: Protect Your Benefits with a Special Needs Trust (SNT)**

If you receive money through an inheritance, gift, legal settlement, or other windfall it can affect your SSI and Medicaid eligibility unless it is placed in a Special Needs Trust.

**What is a Special Needs Trust?**

- A legal account that holds money for your benefit without counting against your SSI or Medicaid asset limits.
- Funds can be used for supplemental needs for things Medicaid doesn't cover, like recreation, transportation, education, technology, and quality of life expenses.
- You need an attorney experienced in disability law to set one up properly.

**Types of Special Needs Trusts:**

- First-party (self-settled) SNT: Funded with your own money (e.g. from a settlement). Requires Medicaid payback at death.
- Third-party SNT: Funded by a parent, grandparent, or other family member. No Medicaid payback required.
- Pooled Trust: Managed by a nonprofit a lower-cost option that can be set up without a private attorney.

**How to find help:**

- Contact your state's Protection & Advocacy (P&A) organization every state has one. Find yours at acl.gov/programs/aging-and-disability-networks.
- Search for a special needs planning attorney in your area through the Special Needs Alliance: specialneedsalliance.org
- Your hospital's social work department may also be able to connect you with local resources.

**❌ Common Myths — These statements are NOT true**

**✗** You must be wealthy to need a Special Needs Trust.

**✗** SNTs are only for people who receive large inheritances or lawsuit settlements.

**✗** If I set up a Special Needs Trust, I lose control of my money.

**✗** You don't need an SNT if you already get SSI or Medicaid.

**Step 4: Open an ABLE Account**

An ABLE account is a federally established, tax-advantaged savings account for people with disabilities. It allows you to save money without losing SSI or Medicaid- up to $100,000 in an ABLE account does not count against the SSI asset limit.

**What can ABLE funds be used for?**

- Education and job training
- Housing and utilities
- Healthcare and wellness
- Transportation
- Assistive technology and personal support
- Recreation and quality of life expenses

🌐 You can open an ABLE account from any state's program you do NOT have to use your own state's program.

Each state's ABLE program has slightly different investment options, fees, and features. You are free to shop and compare programs across all 50 states and choose the one that best fits your needs.

Start your search at: ablenrc.org, the national ABLE resource center, where you can compare all state programs side by side.

**Key rules to know:**

- Eligibility: You must have a disability that began before age 26 (federal law expanded this from age 26 to 46 — check ablenrc.org for the current age limit as legislation has been updated).
- Annual contribution limit: Up to the federal gift tax exclusion amount per year (currently $18,000 from all sources combined; employed ABLE account holders may contribute additional amounts).
- If your ABLE account balance exceeds $100,000, SSI payments are temporarily suspended but you do not lose eligibility.
- You can have both an ABLE account AND a Special Needs Trust, they serve different purposes and work well together.

**❌ Common Myths — These statements are NOT true**

**✗** I have to open an ABLE account in the state where I live.

**✗** Opening an ABLE account will reduce my SSI automatically.

**✗** ABLE accounts are only for people who work full-time.

**✗** I can't use ABLE funds for housing, transportation, or education.

**✗** You can't have both an ABLE account and a Special Needs Trust.

**✗** If I don't use all my ABLE funds, the government takes everything.

**✗** My child is under 26 — it's too early to set one up.

**Key Takeaways**

**✓** Apply for SSI as soon as you turn 18. Benefits are not retroactive.

**✓** Medicaid waiver waiting lists can be long — apply early, even if you don't need services now.

**✓** Keep your own assets below the SSI limit unless funds are in an SNT or ABLE account.

**✓** You can open an ABLE account from any state — shop for the program with the best fit for you.

**✓** A Special Needs Trust and an ABLE account can work together — they are complementary tools.

**✓** Work with a financial advisor or attorney who has experience with disability planning.

**📞 National Resources**

| **Resource** | **Contact** |
| --- | --- |
| Social Security Administration (SSI) | ssa.gov · 1-800-772-1213 |
| Medicare / Medicaid Information | medicaid.gov · 1-800-633-4227 |
| HCBS Waiver Finder | medicaid.gov/medicaid/hcbs |
| ABLE National Resource Center | ablenrc.org (compare all state programs) |
| Special Needs Alliance (SNT attorneys) | specialneedsalliance.org |
| Disability Rights / P&A Organizations | acl.gov — search by state |
| Benefits.gov (all federal programs) | benefits.gov |

*This resource is for informational purposes only and does not constitute legal or financial advice. Eligibility rules, contribution limits, and program availability may change. Consult a qualified attorney or financial advisor for guidance specific to your situation.*
